# Lateral Spacing of TiO_2_ Nanotube Coatings Modulates In Vivo Early New Bone Formation

**DOI:** 10.3390/biomimetics10020081

**Published:** 2025-01-28

**Authors:** Andreea Mariana Negrescu, Iuliana Ionascu, Madalina Georgiana Necula, Niculae Tudor, Maksim Kamaleev, Otilia Zarnescu, Anca Mazare, Patrik Schmuki, Anisoara Cimpean

**Affiliations:** 1 Department of Biochemistry and Molecular Biology, Faculty of Biology, University of Bucharest, 91-95 Spl. Independentei, 050657 Bucharest, Romania; andreea-mariana.negrescu@bio.unibuc.ro (A.M.N.); necula.madalina92@gmail.com (M.G.N.); otilia.zarnescu@bio.unibuc.ro (O.Z.); 2 Research Institute of the University of Bucharest (ICUB), University of Bucharest, 050657 Bucharest, Romania; 3 Faculty of Veterinary Medicine, University of Agronomic Sciences and Veterinary Medicine, 105 Spl. Independentei, 050097 Bucharest, Romania; iuliana.ionascu@usamv.ro (I.I.); niculae.tudor@fmvb.usamv.ro (N.T.); 4 Department of Materials Science and Engineering, Chair of General Materials Properties, Friedrich-Alexander-University of Erlangen-Nürnberg, Martensstraße 5, 91058 Erlangen, Germany; kamaleev.maks@gmail.com; 5 Department of Materials Science and Engineering, WW4-LKO, Friedrich-Alexander-University of Erlangen-Nürnberg, Martensstrasse 7, 91058 Erlangen, Germany; schmuki@ww.uni-erlangen.de; 6 Regional Centre of Advanced Technologies and Materials, Šlechtitelů 27, 78371 Olomouc, Czech Republic

**Keywords:** nanotopographic surfaces, TiO_2_ nanotubes, intertube spacing, osteoblasts, in vivo new bone formation

## Abstract

Due to the bio-inert nature of titanium (Ti) and subsequent accompanying chronic inflammatory response, an implant’s stability and function can be significantly affected, which is why various surface modifications have been employed, including the deposition of titanium oxide (TiO_2_) nanotubes (TNTs) onto the native surface through the anodic oxidation method. While the influence of nanotube diameter on cell behaviour and osteogenesis is very well documented, information regarding the effects of nanotube lateral spacing on the in vivo new bone formation process is insufficient and hard to find. Considering this, the present study’s aim was to evaluate the mechanical properties and the osteogenic ability of two types of TNTs-based pins with different lateral spacing, e.g., 25 nm (TNTs) and 92 nm (spTNTs). The mechanical properties of the TNT-coated implants were characterised from a morphological point of view (tube diameter, spacing, and tube length) using scanning electron microscopy (SEM). In addition, the chemical composition of the implants was evaluated using X-ray photoelectron spectroscopy, while surface roughness and topography were characterised using atomic force microscopy (AFM). Finally, the implants’ hardness and elastic modulus were investigated using nanoindentation measurements. The in vivo new bone formation was histologically evaluated (haematoxylin and eosin—HE staining) at 6 and 30 days post-implantation in a rat model. Mechanical characterisation revealed that the two morphologies presented a similar chemical composition and mechanical strength, but, in terms of surface roughness, the spTNTs exhibited a higher average roughness. The microscopic examination at 1 month post-implantation revealed that spTNTs pins (57.21 ± 34.93) were capable of promoting early new bone tissue formation to a greater extent than the TNTs-coated implants (24.37 ± 6.5), with a difference in the average thickness of the newly formed bone tissue of ~32.84 µm, thus highlighting the importance of this parameter when designing future dental/orthopaedic implants.

## 1. Introduction

With the unprecedented ageing of the world’s population, the need for medical implantable devices suitable for oral rehabilitation and orthopaedic-related traumas/diseases is predicted to grow in accordance with their high demand [1]. With a favourable combination of properties, such as a high mechanical strength approximating the native bone, chemical stability, good biocompatibility and low costs, titanium (Ti) and its alloys continue to be the first-choice type of material in diverse clinical applications for hard tissue replacements [2]. However, in spite of the advantageous properties, the bio-inert nature of the untreated Ti-based biomaterials can compromise initial cell adhesion and the subsequent osseointegration process, with the consequence of implant failure as a possible outcome [3]. In order to overcome this drawback and promote osseointegration, a primary strategy adopted by researchers is the deposition of a functional coating with osteoconductive and osteoinductive properties, characteristics which are vital, especially for biomaterials targeting either dental or orthopaedic bone regeneration [4]. Thus, by modifying the biomaterial’s surface, the long-term performance of the implant can be greatly improved by enhancing the structural and functional connections established between the native bone tissue and the implant’s surface [5]. In addition, surface modification improves the properties of or provides additional functionality to the implantable biomaterial, e.g., better wear and corrosion resistance, favourable biocompatibility and appropriate surface wettability [6].

In the last two decades, nanotopographically altered biomaterials, with nanotopographies within the dimensional range of 100 nm or lower, have been widely evaluated due to their increased biological response and a larger surface area [7]. Amongst the various surface modification techniques, electrochemical anodization is established as one of the most reliable strategies for the synthesis of nanoscale topographies (e.g., TiO_2_ nanotubes) on Ti-based biomaterials in a simple, inexpensive and reproducible manner [8,9]. In addition, this versatile technique enables the formation of highly ordered nanotube features of controlled nano-scale dimensions for an effective biological performance [6]. However, as with other methods, there are a number of challenges, including limited mass production (depending on the anodization conditions) and difficulties in separating the TiO_2_ nanotubular layer from the substrate if individual nanoparticle structures are desired due to the fact that the nanostructures are grown directly on the Ti metal and a limited number of chemical compounds are able to chemically dissolve Ti [9]. Moreover, because the optimisation conditions can greatly influence the properties of the resulting TNTs, the pre-surface characteristics, voltage ramp, fluoride ion concentration and nature of the used electrolyte are also of major concern [9,10] when choosing the synthesis parameters in relation to the desired morphology and final application. In view of the desired nanotopography, control over the nanotube diameter and length is needed or using a classical close-packed or spaced TNTs structure should be considered, where the latter show individual spacing between nanotubes. While anodization itself is advantageous as the coating is directly grown on the metallic substrate, in the case of biomedical applications, the challenges encountered are typically related to (i) the burst release when targeting drug-delivery applications; (ii) additional mechanical strength characterisation being needed for TiO_2_ nanotubes with different nanotube densities (i.e., spaced TNTs); (iii) topography of the nanostructure at the top surface significantly influencing cell interactions [11]; and (iv) previous anodization conditions possibly not leading to similar nanotopographies when translating the anodized layer from flat Ti substrates to Ti pins or screws for in vivo experiments, and thus the anodization conditions needing to be further screened.

Nevertheless, despite the aforementioned challenges, anodic TiO_2_ nanotubes have been the main point of focus in numerous in vitro studies, mainly due to their outstanding characteristics, such as excellent biocompatibility, mechanical rigidity, control over diameter size, uniformity and long-range ordering, high surface area, high loading capability and chemical stability [9], properties which have been proven to favour bone cell adhesion, proliferation and differentiation [3,12]. Moreover, in vitro studies have demonstrated that the textured Ti surfaces can provide larger bone implant contact areas and an improved bone bonding strength in comparison to smoother surfaces, leading to a more favourable in vivo osseointegration process [13]. For example, Bjusten et al. [13] investigated the in vivo bone-bonding strength of two different nanotubular Ti surfaces, namely, TiO_2_ NTs and TiO_2_ grit-blasted surfaces, and the reported results indicated that after four weeks of implantation in rabbit tibias, the nanotubular surface was capable of improving the bone-bonding strength by as much as nine-fold when compared to the Ti grit-blasted implant. Likewise, the histological analysis revealed a larger bone-implant contact area and an improved new bone formation on the TiO_2_-NT modified surface. In another in vivo study, von Wilmowsky et al. [14] compared the osteogenic potential of a TiO_2_ nanostructured implant consisting of nanotubes with a 30 nm tube diameter with that of an untreated standard Ti surface. The immunohistochemistry analysis revealed a higher collagen type 1 expression starting from the 7th day post-implantation up until the 30th for the nanostructured implants in comparison to the control group, suggesting that these implants could influence new bone formation by enhancing the osteoblast function in the early stage of bone development. Similar results were also obtained by Kang et al. [15], where the osseointegration potential of several TiO_2_ nanotubular implants with various diameters (30 nm, 70 nm and 100 nm) was compared to that of an untreated flat Ti surface. Overall, the results proved the superiority of the TiO_2_ nanotubular surfaces in regard to the osseointegration process when compared to the untreated surface, but when analysed from a time point of view, it became clear that between the three modified surfaces, only the TiO_2_ nanotubes with diameters of 30 nm and 70 nm were capable of improving new bone formation and promote implant tissue integration. Moreover, in another study, the effects of similarly sized TiO_2_ nanotubes, i.e., diameters of 30 nm, 70 nm, and 100 nm, on the in vivo osseointegration process were investigated by studying the gene expression and new bone formation in the vicinity of the implants. Compared to the machined Ti implant, the TiO_2_ nanostructured surfaces, especially with a TNT diameter of 70 nm, led to a significant increase in bone-implant contact area and gene expression levels [16]. Furthermore, the beneficial effects of the 70 nm TNT diameter were also confirmed by Jang et al. [17], where miniscrews coated with 70 nm diameter TNTs were implanted in the legs of New Zealand white rabbits for 8 weeks. The histological analysis revealed that the miniscrews with the modified surface showed not only a greater mean bone–implant contact area (52.8%) than the control (29.3%), but also a mean bone volume ratio with an 81% increase. Concerning the influence of the surface chemistry and crystallinity of the TiO_2_ nanotubes, Park et al. [18] showed that the nanoscale geometry clearly dominates surface chemistry or crystallinity, an observation that is also valid for other type of oxides such as ZrO_2_ [9].

As a nanomaterial with multidimensional regulatory potential, TiO_2_ NTs exhibit a series of tuneable parameters. However, upon a comprehensive analysis of the specialised literature, it was revealed that the majority of the existing studies have as their primary objective the evaluation of the influence of TNT diameter on the in vitro behaviour of bone-derived cells [18,19,20,21] and in vivo implant osseointegration [13,14,15,16,17,22,23,24]. On the other hand, little effort has been made to understand how other TNT topographical characteristics, such as wall thickness, length, and lateral spacing, can modulate the cellular response. In an attempt to bring novelty to an area of research overrun by diameter studies and encouraged by the positive results reported on other nanostructures such as nanorods [25,26,27] and nanopillars [28,29], our team designed and investigated, in a previous in vitro study, the behaviour of the MC3T3-E1 cells grown onto the surface of two TiO_2_ nanotubular structures exhibiting different intertube spacing, namely, 18 nm and 80 nm [12]. It was concluded that this parameter can indeed modulate the in vitro behaviour of this pre-osteoblast cell line, inducing a more pronounced osteogenic activity. However, in order for the in vitro findings to be further validated, an in vivo study investigating the direct contact between the native bone tissue and the newly designed implant surfaces was necessary. For this reason, in the present study, two different types of anodic TiO_2_ nanotubes were obtained, more specifically, typical close-packed nanotubes or TNTs, and spaced nanotubes or spTNTs. The nanostructures were grown onto the surface of Ti metal and Ti-based pins, and as a first objective, their anodization parameters were optimised to ensure similar morphological parameters on both substrates in terms of tube diameter, spacing, and tube length. In addition, their chemical composition (X-ray photoelectron spectroscopy), surface roughness and topography, as well as hardness and elastic modulus (nanoindentation measurements), were also evaluated. The second objective was to investigate the effects of the newly developed TiO_2_ nanotubes on the early in vivo new bone formation via the histological analysis of bone sections harvested from the animal subjects at 6 and 30 days post-implantation.

Since the demand for dental and orthopaedic implants will not slow down in the coming years, mainly due to the ever-growing ageing population and the increasing prevalence of chronic diseases, the need for advancements in this field has never been more important [30]. Improvements in implant technology are imperative not only to enhance the durability and effectiveness of the devices, but also to address the intricate challenges associated with biocompatibility, host immune responses, and inadequate new bone formation and osseointegration [31]. The present study, which, to our knowledge, is the first of its kind, demonstrates the superiority of the newly designed spTNTs over the conventional close-packed TNTs with respect to both mechanical properties and early in vivo new bone tissue formation (1 month post-implantation), suggesting that lateral spacing is a parameter worthy of consideration when designing future TiO_2_-based implants for dental/orthopaedic bone regeneration. This can lead to the design of next-generation implants that are not only safe in their interaction with the surrounding microenvironment but are also effective in influencing cellular processes to enhance new bone formation, reduce treatment time and promote a more rapid return to function. Such implants have the potential to significantly improve patient outcomes by providing more effective medical devices that meet both the technical requirements and the clinical needs of the patient.

## 2. Materials and Methods

### 2.1. Implant Preparation and Characterisation

**Scheme 1:**
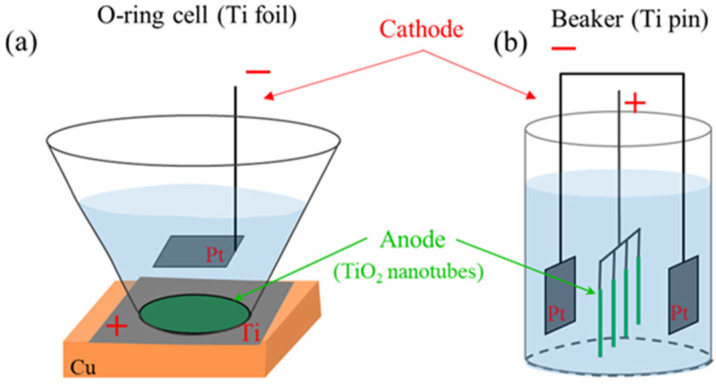
Schematic drawing of the anodization setup for nanotubes grown on Ti foil vs. pins: (**a**) O-ring cell, with the placement of the working sample (anode, Ti foil) and the Pt electrode, and (**b**) beaker, showing the placement of the Ti pins and of the two Pt electrodes.

For control and additional material characterisation, the tubes on foils were also obtained following the previously established anodization conditions [12,32]. Briefly, for close-packed nanotubes, Glycerol (>99.7% p.a. Roth, Karlsruhe, Germany)/H_2_O (70:30 vol.%) + 0.5 wt.% NH_4_F (>98% p.a. Roth, Karlsruhe, Germany) at 21 V for 2 h was used, and for spaced nanotubes, Diethylene glycol (>99.5% p.a. Roth, Karlsruhe, Germany) + 4 wt.% HF (HF 40%, Sigma Aldrich, Germany) + 0.3 wt.% NH_4_F + 7 wt.% H_2_O, at 27 V, 4 h at 30 °C was used. The TiO_2_ nanotubes were obtained in an O-ring cell with a diameter of 2 cm, on a Ti foil which was cut in a 2.5 × 2.5 cm size. An overview of the anodization schematic for anodization performed for nanotubes on foil or on the pin is shown in Scheme 1.

The in vivo animal studies were performed with Ti-based pins with a diameter of 1 mm and a length of 16 mm. The Ti pins were cut from Ti wire (Ti 99.6% purity) initially with a 22 mm length for use in the anodization process, and after the anodization process, the Ti pins were cut to 16 mm length. The implants were ultrasonicated in acetone, ethanol for 5 min each, followed by rinsing with distilled water and drying in a nitrogen stream. The Ti pins were chemically etched for 2 min in a chemical etching solution consisting of 5M HNO_3_ and 40 g/L HF. After etching, the pins were rinsed with distilled water, dried in a nitrogen stream, and used as the working electrode in the electrochemical anodization. Compared to the nanotube layers grown on Ti foil, for the Ti pins, the anodization conditions were optimised. Anodization was performed in a beaker, using two Pt electrodes as the cathode and Ti pins as an anode. The anode consisted of four Ti pins (2.2 cm) welded on a Ti wire, and the distance of the anode to the corresponding Pt electrode was of 2 cm. The close-packed nanotubes were obtained by anodizing the Ti pins in an electrolyte containing Glycerol/H_2_O (70:30 vol.%) + 0.5 wt.% NH_4_F, at 19.5 V, for 1 h 45 min, at room temperature, using 45 mL electrolyte. The spaced nanotubes were obtained in an electrolyte containing Diethylene glycol + 2 wt.% HF (HF 40%) + 0.3 wt.% NH_4_F + 1 wt.% H_2_O, at 77 V for 4 h, using 115 mL of electrolyte. After anodization, the nanotubes were washed with water and dried in a nitrogen stream, except for the spaced nanotubes, which were first immersed in ethanol for 20 min.

The morphology of the nanotubular layers in terms of tube diameter, spacing, and tube length was evaluated by means of a scanning electron microscope (SEM, FE-SEM 4800SEM, Hitachi, Tokyo, Japan). A total of three different samples were measured, with checks being performed in several locations for each. The chemical composition was evaluated by X-ray photoelectron spectroscopy (XPS, PHI 5600, RBD Instruments, Inc., Bend, OR, USA) by sputtering with Ar+ sputtering (3 nm/min calibrated to Si/SiO_2_).

These nanotube layers were used for further characterisation, including SEM, atomic force microscopy (AFM), and nanoindentation measurements. The morphology of the nanotubular layers on pins was evaluated similarly to that of the nanotubes on foils. AFM measurements were performed on Agilent LS5600 (Agilent Technologies, Santa Clara, CA, USA), using cantilevers SSS-SEIHR-10 in tapping mode. Images were taken in duplicate. Nanoindentation measurements were performed with a Nanoindenter XP (Keysight, Colorado Springs, CO, USA), using a diamond Berkovich tip (Synton-MDP, Nidau, Switzerland) coupled with the Continuous Stiffness Measurement (CSM) option and 450 nm depth indents. The samples were fixed on the aluminium holder by a Cristall Bond thermal adhesive (Crystal Bond™), allowing for minimal mechanical vibrations and thermal drift during the measurements. Briefly, the samples and holders were first cleaned with isopropyl alcohol (to remove contaminants and ensure optimal adhesion). Then, the holder was heated to ~150 °C and the thermal adhesive was applied onto the holder by melting the solid stick upon contact with the surface, followed by the placement of the samples. The resulting fixed samples were cooled to room temperature to allow the adhesive to cure and attach. Measurements were performed at room temperature after a three-hour pre-holding of the indenter tip on the sample surface to stabilise the temperature (achieving a thermal drift value ≤ 0.05 nm/s). At least eight indents from each morphology (including the two nanostructured layers and the bare Ti) were used for evaluating the hardness and elastic modulus. The distance between the centres of neighbouring indentations was 10 µm, 22 times higher than the maximum penetration depth employed, ensuring high accuracy of local mechanical properties and reliability (no overlap of the plastic zones formed around the contact points).

### 2.2. Animals

In the current study, a total of 8 healthy adult male Wistar rats with a body weight of 0.25 kg were randomly divided into two experimental groups (4 animals per group), according to the experimental time period. In order to eliminate individual variability, the Ti-based pins were inserted into each hind leg, e.g., TNTs, right leg, and spTNTs, left leg. Prior to the surgical procedure, the animals were kept in acclimatised cages for 2 weeks, where their food and water intake were carefully monitored. All of the animal experiments were approved by the Bioethics Committee of the University of Agronomic Sciences and Veterinary Medicine of Bucharest (Approval code No. 07/28.06.2016) and were conducted according to the international, national and institutional guidelines for the care and use of animals.

### 2.3. Surgical Procedure and Post-Operative Care

**Figure 1:**
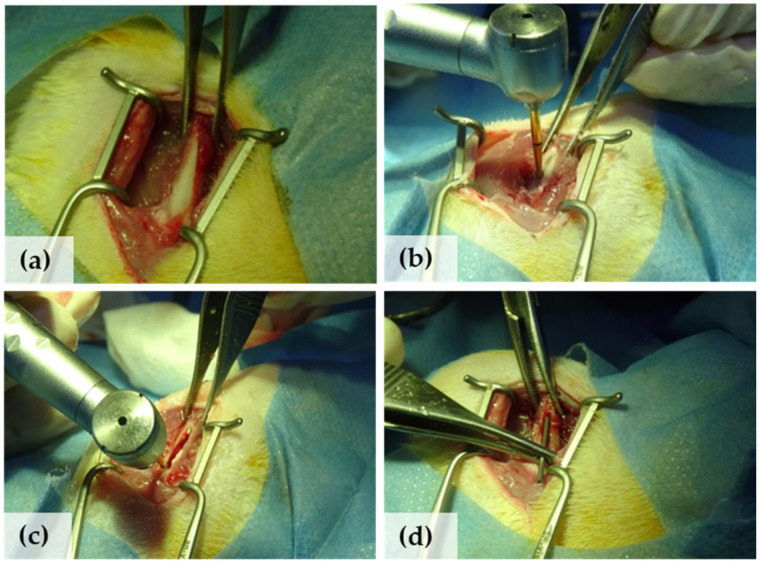
Representative photographs showing the bone implant surgical procedure: (**a**) longitudinal incision on the outer side of the thigh to cut the skin and dissect the underlying muscle tissue; (**b**,**c**) longitudinal fracture transverse to the middle third of the femoral diaphysis; (**d**) implant insertion.

**Figure 2:**
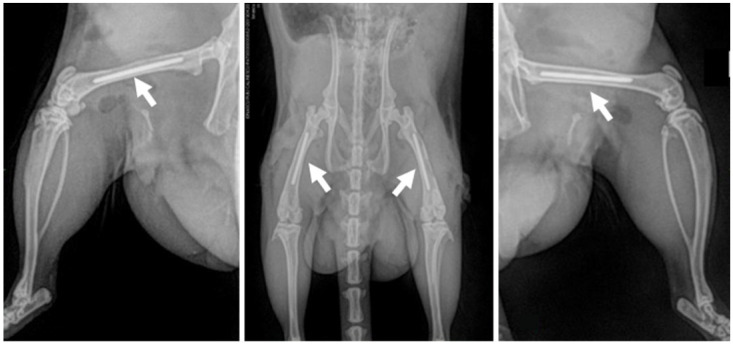
The X-ray analysis of the areas subjected to surgery immediately after the medical intervention confirming the intramedullary presence of the Ti-based implants at the level of the left and right femurs, as indicated by the white arrows.

To achieve the anaesthetic state necessary for the surgical procedure, the animals were subjected to intraperitoneal administration of an anaesthetic mixture containing 100 mg/mL ketamine (1.2 mL) and 0.5 mg/mL dexdomitor (0.8 mL) in 2 mL of saline solution. Moreover, the sustained anaesthetic state was accomplished by mask inhalation of isoflurane vapourised at concentrations of 1–1.5 vol.%. Once the animals were put under general anaesthesia and the surgical area was shaved and disinfected, a 3 cm longitudinal incision on the external side of the thigh was made in order to cut open the skin and dissect the muscle tissue found beneath (Figure 1a). Afterwards, using a Dental Unit cutter, a 2 cm long, linear, longitudinal fracture was created transversally in the middle third of the femur’s diaphysis (Figure 1b,c) and the implants were inserted intramedullary (in both posterior legs) through the newly generated fracture (Figure 1d). In the end, the thigh muscle was sutured with absorbable polydioxanone (PDS 3/0) threads in separate points, whereas the skin above was sutured with nylon (4/0) threads in a simple continuous pattern. To confirm the position of the implants, an X-ray analysis of the areas subjected to surgery was performed immediately after this intervention (Figure 2). With regard to post-operative care, the animals were given antibiotics (Enrofloxacin—10 mg/kg BW) and anti-inflammatory drugs (Meloxicam—0.2 mg/kg BW) daily, for a period of 6 days. Furthermore, no morbidity or mortality was recorded during the 6 and 30 days of the experimental period and the surgical incision was left to heal naturally without any medical intervention.

### 2.4. Post-Operative Tissue Harvesting

At 6 and 30 days post-implantation, the animals were subjected to the same premedication as in Section 2.3 in order to achieve the anaesthetic state necessary for the surgical removal of the bone specimens. Following anaesthesia, the skin was sectioned on the external side of the thigh, in a longitudinal incision of 3 cm, which allowed for the removal of the bilateral femurs, together with the surrounding tissue, by disarticulation. In the end, the animals were euthanised by intraperitoneal administration of 0.5 mL of T61.

### 2.5. Histological Examination

Immediately after euthanasia, the harvested bone samples were firstly fixed for 72 h in a 4% buffered formaldehyde solution, decalcified for 8 days in 5% nitric acid solution, washed for 24 h in 5% solution of sodium sulphate, and only when the bone structures became soft were the specimens embedded in paraffin wax. Sections of 8 µm in thickness were obtained using a rotary microtome (MICROS Produktions-und HandelsgesmbH, St. Veit an der Glan, Carinthia, Austria) and for the histological examination, the Alcian Blue (pH 2.5) and haematoxylin and eosin (HE) staining methods were employed. The photomicrographs were taken by a digital camera (AxioCam MRc 5; Carl Zeiss, Oberkochen, Baden-Württemberg, Germany) driven by software AxioVision 4.6 (Carl Zeiss, Oberkochen, Baden-Württemberg, Germany). Moreover, in order to assess the thickness of the newly formed bone tissue, the distance between the outer and the inner surfaces of the bone tissue was measured using the Axio-Vision software, Version 4.6 (Carl Zeiss, Oberkochen, Germany). The wall thickness was measured at 6 random points and the mean thickness of each newly formed bone tissue was compared.

### 2.6. Statistical Analysis

Means and standard deviations (SDs) were calculated for all datasets. Data from newly formed bone tissue measurement were expressed as the mean of six measurements. Software GraphPad Prism 6 (GraphPad Software Inc., San Diego, CA, USA) was used for data interpretation. Student’s T-test was used to compare the difference between two groups. Significant statistical differences were considered at *p* < 0.05.

## 3. Results and Discussions

### 3.1. TiO_2_ Nanotubes

**Figure 3:**
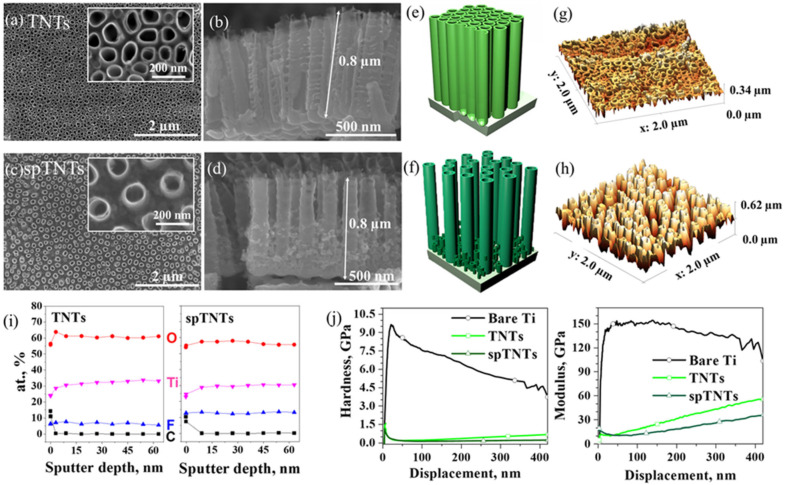
Anodic TiO_2_ nanotubes obtained on Ti metal foil: (**a**–**d**) SEM images of the anodic layers for (**a**,**b**) TNTs and (**c**,**d**) spTNTs, including cross-section SEM image showing the thickness of the layers; (**e**,**f**) corresponding 3D schematic of TNTs and spTNTs, respectively; (**g**,**h**) corresponding 3D AFM images for the TNTs and spTNTs. (**i**) XPS sputter depth profiles showing the at.% composition in the first 60 nm of the layers, namely C, F, Ti, and O. (**j**) Nanoindentation measurements for bare Ti (as reference) and the two different nanotube layers (TNTs, spTNTs), with hardness (**left panel**) and elastic modulus (**right panel**).

In the present work, we tailored two different types of anodic TiO_2_ nanotubes, namely, the typical close-packed nanotubes, or TNTs (such layers have only some minor spacing at the tube top), and the spaced nanotubes, or spTNTs, with a distinct tube-to-tube spacing which is directly proportional to the nanotube diameter, as schematically shown in Figure 3a–f. Such nanotubes are typically grown in fluoride-containing organic electrolytes based on diethylene glycol, dimethyl sulfoxide, etc. [12,33], and the corresponding nanotube layers grown on Ti foil are evaluated in Figure 3.

From the SEM images in Figure 3a–d, the nanostructures have similar morphology, except for the spacing in between nanotubes, which is varied. The obtained classical TNTs have an average tube diameter of 79.4 nm (±14.2 nm) and a tube spacing at the tube tops of 22.4 nm (±5.9 nm). In contrast, the spTNTs have a tube diameter in a similar range (83.1 nm ± 17.1 nm) and a spacing of 83.5 nm (±11.6 nm). Both nanotube layers have an average tube length of 0.8 µm. The chemical composition of such tubes is similar to the previously obtained morphologies [12,32], and consists of C, O, Ti and F; the at.% compositions shown in the sputter-depth profiles (first 60 nm of the layers—Figure 3i) confirm the TiO_2_ nature of the oxide with ≈6.5 at.% F and ≈11.5 at.% F for the TNTs and spTNTs, respectively.

Furthermore, the surface roughness and topography of the layers was evaluated through AFM (Figure 3g,h). The 3D AFM images clearly show the topographical differences between the two nanostructures (with a root mean-square roughness, Rq, of 42.06 nm and 119.9 nm, respectively). The corresponding line profiles (average of three individual profiles) show that the TNTs have a height of up to 0.3 µm and spTNTs have a height of almost 0.6 µm.

Since the nanostructured layers need to be mechanically durable in order to be used as biomedical implants, we performed nanoindentation measurements on bare Ti (the bare Ti sample was measured as a reference) and the studied layers (Figure 3j). As shown in Figure 3j (left panel), the bare Ti sample shows a hardness of ≈4.5–8.5 GPa, while for the nanotube layers, if we consider the values up to 80 nm displacement (which is less than 10% of the total height of the nanotubes, and so has no influence from the substrate) [34], the hardness reaches ≈0.5–1.0 GPa. The elastic modulus of Ti is ≈120–150 GPa, while for the nanotube layers (similarly at 80 nm displacement), the elastic modulus is ≈10–20 GPa. The observed decreases can be attributed to the nanostructure formation, and with increasing the displacement, more influence from the substrate can be observed.

**Figure 4:**
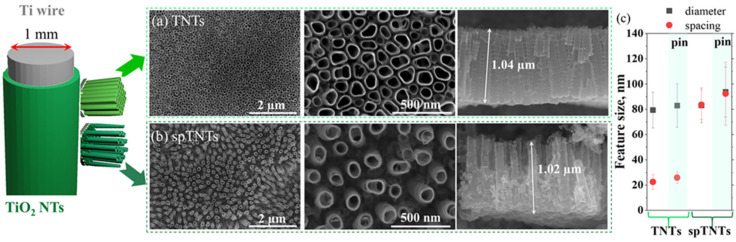
Anodic TiO_2_ nanotubes obtained on Ti pins for in vivo tests: schematic representation and corresponding SEM images of the anodic layers for (**a**) TNTs and (**b**) spTNTs. The SEM images include lower magnification to show the layer uniformity (**left panel**), higher magnification to observe the tube morphology (**middle panel**), and cross-section images showing the length of the nanotube coatings (**right panel**). (**c**) Feature size (diameter, spacing) of the designed TNTs and spTNTs, depending on the substrate (foil or pin).

Furthermore, similar morphology nanotube layers were grown on Ti pins with diameters of 1 mm (as shown schematically in Figure 4), and for this reason, the anodization conditions were optimised even more for the spTNTs (for the detailed optimised conditions, please see the experimental section). Figure 4a,b shows the tube morphology of the layers grown on pins, with tube layers with 1.0 µm thickness. The average diameter of the layers is ≈82–92 nm, namely, 83 nm ± 17 nm for the TNTs and 91 nm ± 19 nm spTNTs, but with different spacing of 25 nm ± 4 nm for TNTs and 92 nm ± 25 nm for spTNTs. An overview of feature size (diameter, spacing) as a function of the used substrate (foil or pin) is listed in Figure 4c.

### 3.2. The Histological Appearance of the Post-Implant Tissue

Since the histological characterisation of the osseointegration process is crucial for a more in-depth understanding of the intimate relationship established between the damaged bone tissue, the implant, and the ability of the material to support new bone formation, the short-term in vivo evaluation of the biological behaviour of two nanostructured Ti-based implants (TNTs and spTNTs) was assessed through the histological analysis of bone sections harvested from the animal subjects at 6 and 30 days post-implantation. The rationale behind the selection of the 6- and 30-day post-implantation periods is to facilitate the evaluation of the early formation process and growth trend of the new bone tissue [35,36]. This approach enables the observation of the osteogenic promotion effect of the spTNTs pins in comparison to the TNTs implants.

**Figure 5:**
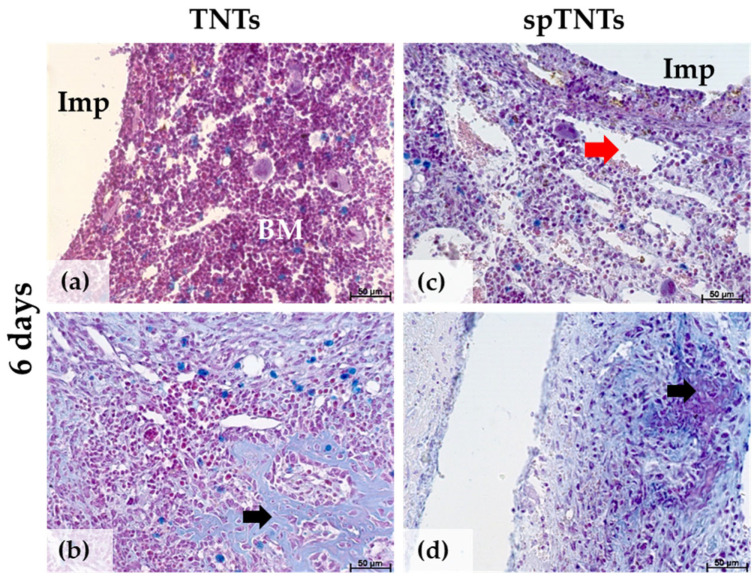
Haematoxylin–eosin (HE) staining of bone sections surrounding the Ti-based implants: (**a**,**b**) TNTs and (**c**,**d**) spTNTs, harvested 6 days after the surgical intervention. BM, bone marrow; Imp, intra-medullary implant site. Red arrow—altered bone stroma; black arrows—unmineralised bone tissue (osteoid) secreted by osteoblasts. Scale bar: 50 µm.

The histological analysis of the peri-implant tissue harvested 6 days post-implantation showed visible dissimilarities between the investigated biomaterials, as seen in Figure 5. Thus, the bone sections from the animal subjects with the modified Ti-based implants highlighted the existence of a thin fibrous tissue adjacent to the peri-implant site in the case of both implants. However, the adjacent bone marrow presented a different aspect depending on the features of the nanotubular TiO_2_ structures generated on the surface of the Ti-based implant, i.e., with the spTNTs pin leading to a slight modification (red arrow) in the bone marrow structure (Figure 5b), as opposed to the TNTs-coated implant, where the structure and cellularity of the bone marrow did not appear to suffer any alterations (Figure 5a). It is important to mention that regardless of bone marrow alteration, in the case of both modified surfaces, the ability of the osteoprogenitor cells to differentiate and that of the osteoblasts to secrete osteoid was not affected, as evidenced by the presence of the osteoid-secreted osteoblasts (black arrows).

**Figure 6:**
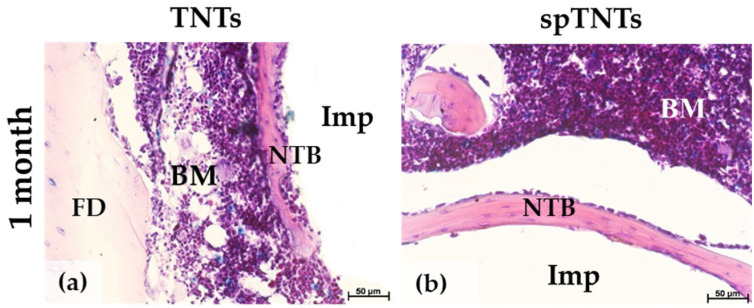
Haematoxylin–eosin (HE) staining of bone tissue surrounding the implants: (**a**) TNTs and (**b**) spTNTs, at 30 days post-implantation. Imp, intra-medullary implant site; BM, bone marrow; FD, femoral diaphysis; NTB, newly formed trabecular bone. Scale bar: 50 µm.

**Figure 7:**
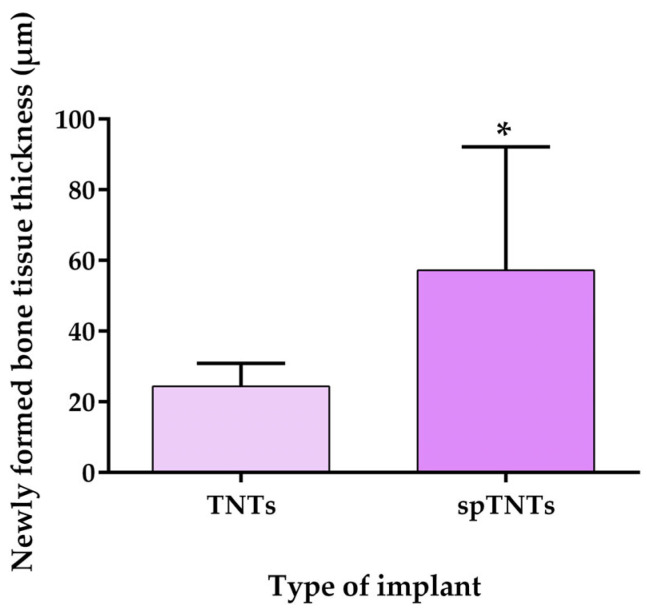
Comparison of newly formed bone tissue thickness (µm) on the transverse histological section of bone (means ± SD) in case of TNTs (24.37 ± 6.5), and spTNTs implants (57.21 ± 34.93) (* *p* < 0.01 vs. TNTs).

In contrast with the results observed at 6 days post-implantation, the histological analysis of the bone sections harvested at 30 days post-implantation highlighted a progressive new bone formation. Thus, Figure 6 reveals the presence of a noticeable compact adherent newly formed bone tissue (NTB) with no evidence of fibrous tissue formation for both Ti-based implants. In addition, the histological measurements of the newly formed tissue revealed that the mean thickness of the bone in the case of the spTNTs pin was significantly higher (*p* < 0.01) than that of the TNTs implant (Figure 7). These results suggest that the spTNTs implant can provide a favourable microenvironment for the osteoblasts to promote the ingrowth of mineralised tissue for an enhanced osseointegration process. Moreover, it is noteworthy that no foreign body reaction was observed in the animals, indicating that the implants are well tolerated.

One of the most important aspects of bone-implant integration is represented by the enhancement in the functional activity of osteoblasts at the peri-implant interface without any fibrotic tissue formation [37]. However, the majority of modern orthopaedic implantable devices present a reduced lifespan due to the establishment of an inappropriate cell–material initial interaction, an event with a key role in the wound-healing process, which is heavily dependent on the specific surface properties of the material (e.g., wettability, roughness, topography, etc.) [38].

As evidenced by data reported in the literature, by modifying the Ti-based implant’s surface through anodic oxidation (formation of titania nanotubes), an enhanced new bone formation and osseointegration was observed. For example, Alves-Rezende et al. [39] demonstrated that, as opposed to a pure Ti implant, the nanostructured TiO_2_ surface led to an improved wettability, an increased osteoblastic cells’ migration and implicitly, and an enhanced bone-to-implant contact (BIC) (Cp-Ti—37.89 ± 0.01% vs. Cp-Ti anodised—58.97 ± 0.10%). Similarly, Wang et al. [40] reported that hydrogenated superhydrophilic TiO_2_ surfaces were able to significantly accelerate the new bone formation and osseointegration processes, both in the early and later stages. In addition, in another study by Tao et al. [1], the in vivo investigations revealed that between the three newly proposed TNTs surfaces with different nanotube diameters (e.g., 30 nm, 70 nm and 110 nm), the TNT-70 implant exhibited the highest potential to improve new bone formation and BIC, as evidenced by the more mature and thicker new bone tissue observed at the peri-implant site. Furthermore, apart from the advantageous surface characteristics, the anodic TiO_2_ nanotubes can also be used as drug delivery platforms for various bioactive elements, molecules and compounds capable of promoting bone cell differentiation and new tissue formation [41]. By modifying the surface of a Ti screw with silicon (Si)-doped TiO_2_ nanotubes, the histological analysis revealed an improved new bone formation and a superior bone–implant bonding strength [42]. In another study, a gallium oxide (Ga_2_O_3_) coated TNT drug delivery platform was designed based on the hypothesis that the release of Ga^3+^ ions in a sustainable manner could enhance the osteogenic ability of the Ti-based implant. The in vivo analysis demonstrated that the newly fabricated construct was capable of promoting new bone formation through an enhancement in the osteogenesis-related gene expression [43]. Similarly, the incorporation of platinum (Pt) [44] and strontium (Sr) [45] into TNTs facilitated an efficient mineralisation and led to the creation of surfaces with a higher interfacial bonding strength.

Starting from the hypothesis that the lateral spacing of the TiO_2_ nanotubes can contribute to the continuous flow of the cell culture medium in an in vitro microenvironment, as well as the exchange of gases, nutrients and signalling molecules, thus stimulating the cellular metabolism and better mimicking the in vivo conditions [22,46], in an initial in vitro study, our team investigated the effect of titania nanotube spacing on the MC3T3-E1 pre-osteoblast cell function with interesting results. Thus, depending on the type of nanostructure, the data obtained revealed different cellular responses in terms of cytomorphology, actin cytoskeleton organisation, cell-spreading area and pattern of focal adhesions, while the osteogenic differentiation assessment underlined the beneficial effect of the nanotubes with an intertube spacing of 80 nm [12]. Moreover, the recently established field of osteoimmunology points out that the in vivo osteogenic process is not simply accomplished by the bone-derived cells alone, but through a close collaboration between the skeletal and the immune systems [47]. Therefore, we can now state that the success of the biomaterial’s integration depends heavily on the inflammatory-driven process located at and adjacent to the implant’s surface [48]. The introduction of an implant into the host’s organism is always associated with a surgical injury, which, on its own, will induce a classical pathophysiological acute inflammatory response. Furthermore, depending on the implant’s characteristics such as shape, size, surface chemistry and morphology, the biomaterial will be recognised by the host’s immune system as a foreign body, therefore resulting in a foreign body reaction (FBR)—a chronic inflammatory state which will hinder the success of the osseointegration process and the implant itself [47]. In this context, in a follow-up in vitro study, our team investigated the effect of TNT interspacing on the inflammatory activity of RAW 264.7 macrophages and the obtained data demonstrated that an 80 nm intertube spacing was capable of reducing the inflammatory activity of macrophages through the modulation of their polarisation state towards an anti-inflammatory profile. Overall, the results of this study proved that intertube spacing can represent a passive strategy for macrophage modulation to obtain an inflammatory activity directed towards bone regeneration and osseointegration rather than bone resorption and implant failure [32].

Altogether, these two previous in vitro studies brought forward a new surface feature worthy of more in-depth investigation, which is why, in the current study, the in vivo effect of lateral spacing on the cell–surface interactions and early new bone formation was evaluated. The HE staining of the bone tissue surrounding the implants revealed that the spaced nanotubes were capable of influencing the early osteogenic process (1 month post-implantation) in a positive manner, as demonstrated by the thicker bone tissue formed adjacent to the spTNT pin (57.21 ± 34.93) as compared to the TNTs-coated implant (24.37 ± 6.5) (Figure 6 and Figure 7). The in vivo results obtained are in line with the previously reported in vitro findings, where the spTNTs exhibited a beneficial effect on the pre-osteoblasts’ osteogenic differentiation, evidenced by the increased ALP activity and osteopontin and osteocalcin protein expression [12]. Taken together, our observations indicate that an intertube spacing of ≈90 nm represents a favourable dimension for an accelerated incipient new bone formation. The phenomenon could be attributed to the special nanostructure of the implant’s surface, which combines the positive effect of nanoscale features with an optimal lateral spacing for the regulation of the molecular and cellular activities, which in turn, through various direct and indirect mechanisms, will modulate the osseointegration process [16,49,50].

As mentioned before, implant osseointegration is a complex process that involves multiple biological processes that can be regulated by different implant surface topographical characteristics [51]. Thus, in order to understand how TiO_2_ nanotubular surfaces interact with the adjacent bone tissue, according to the different pivotal biological process, osseointegration can be divided into four phases, namely, protein adsorption, inflammatory response (inflammatory cell adhesion), additional relevant cells’ adhesion and osteogenesis [35,51,52]. The first stage occurs immediately after the implant comes into contact with the adjacent bone tissue and involves the absorption of blood proteins onto the hydrated titanium surface (water molecule absorption occurs several nanoseconds after implant placement), leading to the formation of a “protein layer” which is vital for the subsequent biological process [53]. From data reported in literature, it became clear that these proteins found at the interface between the implant’s surface and cells have extracellular signalling roles in the cytoskeleton organisation [54], thus cementing the idea that TiO_2_ NTs surface characteristics are fundamental and can determine the subsequent cell attachment and spreading [37]. Within a few hours, immune cells and platelets migrate to the implantation situs, and initiate the inflammatory response. In this stage, neutrophils and macrophages are recruited to the implant’s surface to clean the wound site from debris, bacteria and damaged tissue [55]. Moreover, the recruited macrophages release inflammatory mediators such as cytokines, chemokines and growth factors in the microenvironment, fundamental in the subsequent healing process [35]. Therefore, the immunomodulatory nature of the TiO_2_ nanotubes is another aspect that should be taken into consideration when designing new bone implants. In this regard, our team has already demonstrated that spTNTs are capable of reducing the inflammatory activity of macrophages [32], thus indicating that intertube spacing can endow implants with an immunomodulatory function. In the following stage, tissue-repairing cells such as mesenchymal stem cells (MSCs), osteoblasts and fibroblasts are recruited in response to the inflammatory mediators secreted by the immune cells. It is a well-known fact that cells can sense and respond to various extracellular matrix (ECM) biochemical and biophysical environmental cues such as nanotopography [51]. Therefore, shortly after migration, the tissue-regenerating cells are capable of adapting to the nanotopographical characteristics of the surface and recognise the absorbed proteins (e.g., fibronectins, collagens, vitronectins, laminins, etc.) through cell transmembrane proteins that function as ECM–cytoskeleton linkers, i.e., integrins [53,56]. Following the binding of integrins to the targeted ECM proteins, the induction of specific intracellular signalling pathways leads integrins to assemble at the plasma membrane and change their conformation [57]. Subsequently, the integrin assembling promotes various cytoplasmatic proteins and signalling molecules to arrive at the attachment situs, leading to an enhanced adhesion strength and the formation of focal adhesions [51]. As Sun et al. suggested [58], the connection between the cells and ECM through focal adhesions forms a “gear box” capable of perceiving extracellular mechanical forces and achieving mechanotransduction. Data reported in the literature suggest that the TiO_2_ NTs surfaces can affect the MSCs and osteoblasts’ adhesion by providing anchoring sites for cell attachment [59,60], therefore laying the foundation for the subsequent contact osteogenic process and implicitly for a favourable osseointegration. Once the cells have attached, the TiO_2_ NT surfaces can manipulate osteogenic differentiation by playing the role of an extracellular mechanical signal, which is transmitted into an intracellular signal capable of regulating the cell behaviour [51].

As this study is in its initial phase, it is only possible to hypothesise about the molecular mechanism behind the beneficial effect of lateral spacing on the early osteogenic process. The available data demonstrate that the presence of empty nanotube pore spaces and the gap between neighbouring nanotubes forces protein aggregates to adhere to the top wall surface of the TiO_2_ nanotubes [22], which is why, in order for the MSCs to be able to establish the initial contact with the protein aggregates, they need to stretch and elongate their bodies. Such morphological adaptations may result in cytoskeleton stress and tension, with the size of the nanotube being directly proportional to the degree of stress and tension. Moreover, it is a well-known fact that different types of physical stimuli arising from substrate morphology and topography can enhance stem cells’ differentiation into a specific cell lineage, including osteoblasts [60,61]. Similar observations were also reported for osteoblasts, where an elongated morphology was associated with enhanced osteogenic differentiation as evidenced by the increased levels of ALP, mineral deposition and osteogenesis-related gene expression [12,60,62]. In addition, considering the results of our previous in vitro study, where it was observed that lateral spacing can modulate cell differentiation, we can only assume that the nanotopography-mediated signal transduction pathway involves the FAK/RhoA/YAP cascade, as demonstrated by Zhang et al. [63]. In their study, the large-diameter NTs (90 nm) prevented focal adhesion formation, which blocked FAK/RhoA signalling. This, in turn, led to the export of YAP from the nucleus and the activation of Runx2. As a result, the cells grown on the large-diameter NTs underwent osteogenic differentiation. Kong et al. [64] reported similar results regarding the involvement of YAP in the in vitro and in vivo osteogenic differentiation of MSCs, further confirming the theory according to which lateral spacing modulates cell differentiation through mechanotransductive signals.

Although our study demonstrates the positive effect of intertube spacing on new bone tissue formation, the spTNT-based implants need to be subjected to a more in-depth investigation before the new coating can be translated into clinical situations. Finally, it is important to acknowledge that the present study does have two limitations. Firstly, we evaluated the early new bone formation and the used animal model represented a healthy system; so, it would be of interest to observe the long-term osteogenic and osseointegration capabilities of the spTNT-based pin not only in healthy individuals but also in different compromised-healing conditions (e.g., diabetes mellitus) or in models with poor bone quantities or subjected to local radiations. Secondly, the effect of lateral spacing on the in vivo inflammatory process, a pivotal component of osseointegration that determines the fate of an implant, was not investigated.

## 4. Conclusions

In the present study, two types of Ti-based implants, namely, nanotubes with the typical close-packed (TNTs) or spaced (spTNTs) nanotopographies, were obtained with a similar morphology on both flat Ti (typically used for in vitro tests) and Ti pins (to be used in in vivo tests). The fact that nanotubes with similar morphologies were obtained on the two different substrates (tailoring the anodization parameters for the nanotubes grown on Ti pins) allowed us to link previous in vitro studies with the current in vivo one. The two morphologies showed a similar chemical composition and mechanical strength (hardness and elastic modulus—at a displacement where only parameters for the nanotube layers are observed), and while the spTNTs showed a higher average roughness in AFM, this was expected due to the penetration of the AFM tip between the nanotubes. More importantly, such a morphology (spaced nanotubes) would prove advantageous for building functional hierarchical nanostructures (e.g., decorating with metallic or oxide nanoparticles, growth factors, drugs, etc.), for targeting an increased osseointegration and/or antibacterial behaviour.

The layers were evaluated regarding their potential to promote new bone formation so as to improve the osseointegration process at the implantation site. The histological examination revealed that the nanotubular surfaces did not induce any alterations in the bone marrow’s structure and were capable of stimulating the number of osteoblasts and osteoprogenitor cells found at the endosteum level. Moreover, between the two nanotubular surfaces, it was observed that the spTNT pins were capable of promoting new bone tissue formation to a greater extent than the TNTs surface, with a difference in the average thickness of the newly formed bone tissue of approximately 32.84 µm after 30 days of implantation. From morphological and chemical points of view, the two TiO_2_ nanotubes were similar, i.e., similar tube diameter (80–90 nm), tube length (1 µm), chemical composition, except for the tube spacing, which is ≈25 nm for the close-packed configuration and ≈92 nm for spTNTs. Thus, it stands to reason that the differences encountered in the in vivo tests are directly correlated to this intertubular spacing in the TiO_2_ nanotube morphology. Additionally, further questions raised from these findings include the existence of a minimum spacing of the TNTs that results in the promotion of new bone.

Considering the aspects explored in the current study, it is important to mention that the osseointegration of any implantable medical device represents a very complex process and that the impact of nanotube spacing, although promising in the initial phase of the process, as demonstrated, requires more in-depth investigation. Thus, the next experimental step should focus on the effects of intertube spacing on the long-term osseointegration process. Moreover, the underlying mechanisms through which these nanotubular surfaces can mediate bone regeneration and bone ingrowth are still unclear; therefore, additional studies are necessary in order to fully elucidate the intrinsic molecular mechanisms of these processes. Furthermore, the inflammatory process and its mediation through spTNT-coatings also represents a focus in future research.

## Data Availability

Data sharing is not applicable to this article as no new data were created or analysed in this study.
